# Luminally Acting Agents for Constipation Treatment: A Review Based on Literatures and Patents

**DOI:** 10.3389/fphar.2017.00418

**Published:** 2017-06-30

**Authors:** Hong Yang, Tonghui Ma

**Affiliations:** ^1^Liaoning Provincial Key Laboratory of Biotechnology and Drug Discovery, School of Life Sciences, Liaoning Normal University Dalian, China; ^2^Institute of Traditional Chinese Medicine, Nanjing University of Chinese Medicine Nanjing, China

**Keywords:** constipation, chloride channels, activators, fluid secretion, intestinal motility

## Abstract

Constipation is one of the most frequently reported gastrointestinal (GI) disorders that negatively impacts quality of life and is associated with a significant economic burden to the patients and society. Traditional treatments including lifestyle modification and laxatives are often ineffective in the more severe forms of constipation and over the long term. New medications targeting at intestinal chloride channels and colonic serotonin receptors have been demonstrated effective in recent years. Emerging agents focusing on improving intestinal secretion and/or colonic motility have been shown effective in animal models and even in clinical trials. Recognization of the role of cystic fibrosis transmembrane regulator (CFTR) and calcium-activated chloride channels (CaCCs) in intestine fluid secretion and motility modulation makes CFTR and CaCCs promising molecule targets for anti-constipation therapy. Although there are multiple choices for constipation treatment, there is still a recognized need for new medications in anti-constipation therapy. The present review covers the discovery of luminally acting agents for constipation treatment described in both patents (2011–present) and scientific literatures.

## Introduction

Chronic idiopathic constipation (CIC) is one of the most frequently reported gastrointestinal (GI) disorders in general populations. CIC is a complex symptom characterized by reduction in bowel movements and/or defecatory difficulties, including straining at defecation, hard or lumpy stools, sensation of incomplete evacuation, and sensation of blockage or anorectal obstruction. The prevalence of CIC has been estimated to be ~14% worldwide (Mugie et al., 2011; Sanchez and Bercik, 2011; Vazquez Roque and Bouras, 2015). Constipation negatively impacts quality of life and is associated with a significant economic burden to the patients and society. The annual health care cost for CIC was around $7,500 per patient in United States (Liem et al., 2009; Sanchez and Bercik, 2011; Hoekman and Benninga, 2013; Chu et al., 2014). In China, expenditure for over-the-counter (OTC) laxatives is over 8 billion RMB per year (Chu et al., 2014). It is estimated that the long-term direct medical costs of CIC patients will double those of the populations without constipation in the next 15 years.

Causes of constipation may be classified as primary (also called functional or idiopathic) and secondary (also called organic). Secondary constipation is caused by specific recognizable causes including gastrointestinal disorders, metabolic and endocrine disorders, neurological conditions, congestive cardiac insufficiency, psychogenic disorders, dehydration, and a variety of medications. When no definite cause can be demonstrated, constipation is defined as primary. Primary constipation is usually subdivided into three broad categories: normal-transit constipation (NTC), slow-transit constipation (STC), and evacuation disorders (ED) (Koch et al., 1997; Nyam et al., 1997; Costilla and Foxx-Orenstein, 2014).

NTC, the most common form of primary constipation, is a heterogeneous disorder. Although stool frequency and stool transit through the colon is normal, patients with NTC are subjectively think they are constipated (Ashraf et al., 1996). NTC might be due to a perceived difficulty with evacuation or hard stools. The patients may experience bloating and abdominal pain or discomfort, exhibit increased psychosocial distress, or have increased rectal compliance and reduced rectal sensation (Mertz et al., 1999; Rao, 2003). The pathophysiology of NTC is similar to that of constipation-predominant irritable bowel syndrome (IBS-C) (Rey et al., 2014). Irritable bowel syndrome (IBS), a chronic functional disorder of the GI tract, is characterized by abdominal pain or discomfort in association with change in bowel habits and with sensation of bloating. IBS is subclassified as IBS-C, diarrhea predominant (IBS-D) and IBS with mixed features (IBS-M) according to predominant bowel habit (El-Salhy, 2015). The pathophysiology of IBS is traditionally believed to involve multiple factors such as bowel hypersensitivity, altered bowel motility, inflammation and stress, although the precise mechanisms of the symptoms remain unclear. According to the Rome III Criteria, NTC and IBS-C should be theoretically distinguishable mainly by the presence of abdominal pain or discomfort relieved by defecation (typical of IBS); however, many gastroenterologists question whether a real distinction is possible.

ED is most commonly due to dysfunction of the pelvic floor or anal sphincter, which includes an array of problems in which the normal mechanism for expulsion of feces is disturbed or disrupted. Structural problems, such as rectal prolapse, rectal mucosal intussusception, megarectum, rectocele, enterocele, and the descending perineum syndrome, can lead to functional consequences of defecation (Rao et al., 1998). ED may be also caused by inappropriate or inadequate muscle movements with paradoxical contractions, inadequate relaxation of the pelvic floor, and/or poor propulsion. This kind of defecatory disorder is also called anismus, pelvic-floor dyssynergia, paradoxical pelvic-floor contraction, obstructed constipation, functional rectosigmoid obstruction, the spastic pelvic floor syndrome, and functional fecal retention in childhood.

STC is a motility disorder characterized by markedly increased total bowel transit time. Since there are no diagnostic histologic features that can help to determine the underlying etiology, the condition frustrates not only the patients, gastroenterologists and colorectal surgeons, but also pathologists. Histologic features of colectomy specimens can be completely normal or show only secondary nonspecific changes resulting from chronic constipation or its treatment like melanosis coli, cryptitis, crypt abscess, mucosal prolapse, and pneumatosis coli (Wang, 2015). Typically, there are no remarkable morphologic abnormalities in the nerve plexes, ganglion cells and muscle fibers. Abdominal distension is more common and stool frequency is rarer in STC than in NTC. The cause of STC remains uncertain, but may include insufficient intake of dietary fiber, and intestinal neuronal agenesis resulted from hormonal disorders. STC is found predominantly in women and is associated with esophageal, gastrointestinal, urinary bladder motor abnormalities, galactorrhea, orthostatic hypotension, and other extraintestinal symptoms (Bassotti et al., 1996; Altomare et al., 1999; Knowles and Farrugia, 2011; Andromanakos et al., 2015). There is evidence for absence or reduction in the number of interstitial cells of Cajal (ICCs) in STC. How these various abnormalities contribute to the pathogenesis of STC remains to be unveiled.

The pathophysiology underlying CIC is multifactorial and remains poorly understood. At present, the mainstay of anti-constipation therapy consists of increasing stool bulk, creating an osmotic load, stimulating intestinal contraction as well as softening stool (Menees et al., 2012). Both of the US Food and Drug Administration (FDA)-approved drugs linaclotide and lubiprostone are thought to activate enterocyte chloride channels (Busby et al., 2010; Fei et al., 2010; Castro et al., 2013). Linaclotide, a guanylate cyclase type C (GC-C) receptor agonist, stimulates CFTR-mediated Cl^−^ secretion via elevation of intracellular cGMP level (Tien et al., 1994). Linaclotide can also increase intestinal motility through activation of colonic sensory and motor neurons (Hoekman and Benninga, 2013; Chu et al., 2014). Lubiprostone, a prostaglandin E (PGE) analog, was once regarded to activate type-2 chloride channel (ClC-2)-mediated Cl^−^ secretion in enterocytes, but subsequent studies indicated that lubiprostone activated CFTR chloride channel through E-type prostanoid 4 (EP4) receptors (Norimatsu et al., 2012). Main defects of linaclotide and lubiprostone include limited efficacy and dose-dependent nausea. Plecanatide, a GC-C receptor agonist that has a similar mechanism of action to that of linaclotide, is currently under development for CIC therapy (Camilleri, 2015). In recent years, both pro-secretory (to increase intestinal fluid secretion) and prokinetic (to enhance GI motility) agents have shown efficacies for the treatment of various types of constipation including CIC, opioid-induced constipation (OIC) and IBS-C.

The present review covers patents filed or issued in the last 6 years (2011–present) and published literatures toward luminally acting agents for anti-constipation treatment and focuses on the pharmacology, efficacy and safety profiles of the agents. The patent documents were retrieved from the worldwide database of the European Patent Office.

## Prosecretory agents

Stimulation of intestinal fluid secretion through chloride channel activation has been an emerging and very promising subject for anti-constipation treatment in the past decade. Prosecretory drugs, such as the US FDA-approved drugs lubiprostone and linaclotide and an emerging drug plecanatide, work by activating chloride channel activities in luminal surface of the intestine and thereby enhancing Na^+^ and water secretion. Elobixibat (A3309) that increases stool frequency through enhancing delivery of bile acids to the colon is also regarded as a prosecretory agent (Acosta and Camilleri, 2014).

### Lubiprostone

**Figure 1:**
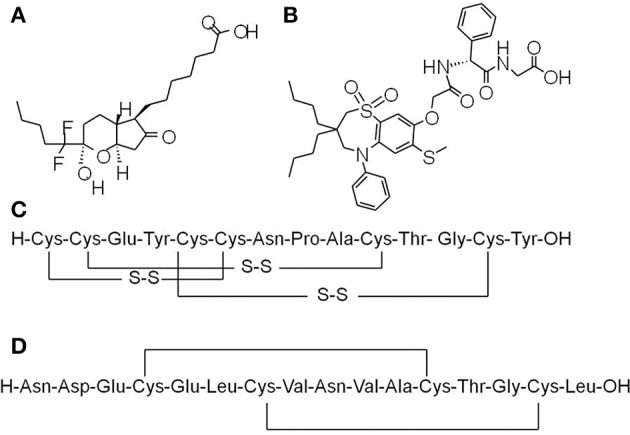
Chemical structures of prosecretory agents. **(A)** Lubiprostone (Amitiza), a bicyclic fatty acid derived from prostaglandin E1 (PGE1), is the first chloride channel-targeted drug that was approved by the US FDA for treatment of constipation. **(B)** Elobixibat, a first-in-class ileal bile acid transporter (IBAT) inhibitor, accelerates colonic transit through enhancing delivery of bile acids to the colon, and thus increasing stool frequency as well as decreasing constipation-related symptoms in chronic idiopathic constipation (CIC) patients. **(C)** Linaclotide, a 14-amino acids peptide homologous to bacterial heat-stable enterotoxins, is a first-in-class anti-constipation drug targeting guanylyl cyclase C (GC-C) receptor on the luminal surface of GI enterocytes. Activation of GC-C receptor increases both intracellular and extracellular levels of cyclic guanosine monophosphate (cGMP). The increase in intracellular cGMP level triggers the activation of cystic fibrosis transmembrane conductance regulator (CFTR) chloride channel activity and thereby results in salt and water secretion into the lumen of intestine. **(D)** Plecanatide, a 16 amino acid GC-C activator, is another synthetic GC-C agonist that activates GC-C receptors located on the luminal surface of intestinal enterocytes, leading to the elevation of intracellular cGMP and subsequent activation of CFTR chloride channel.

Lubiprostone (Amitiza) (Figure 1A) is the first chloride channel-targeted drug that was approved by the US FDA for treatment of constipation in 2006. Lubiprostone, a bicyclic fatty acid derived from prostaglandin E1 (PGE1), works by increasing chloride channel-dependent intraluminal fluid secretion of the intestine (Wilson and Schey, 2015). Lubiprostone was initially thought to selectively activate ClC-2 in the apical membrane of the GI epithelium (Cuppoletti et al., 2004). However, subsequent studies indicated that ClC-2 type chloride channel is mainly located in the basolateral rather than the apical membrane of the enterocytes, where it is involved in the reabsorption of chloride (Catalán et al., 2012). Separate studies showed that lubiprostone stimulated CFTR-dependent chloride secretion via EP4 receptor pathway (Norimatsu et al., 2012) and facilitated internalization of ClC-2 from the basolateral membranes to the cytoplasm (Jakab et al., 2012). Based on these findings, some researchers suggested that lubiprostone works through both increasing CFTR-dependent intraluminal chloride secretion at the apical side and inhibiting ClC-2-dependent chloride reabsorption at the basolateral side (Akiba and Kaunitz, 2012). Lubiprostone has been recently shown to stimulate intestinal circular muscle contraction through EP1 prostaglandin receptor (Jiao et al., 2014). In addition, lubiprostone may also modulate the pacemaking activities of interstitial cells of Cajal (ICCs) located in GI smooth muscle layer (Jiao et al., 2014) as well as decrease small intestinal bacterial overgrowth (Sarosiek et al., 2016). Lubiprostone treatment was well tolerated and improved symptoms and signs of OIC in a 9-month, open-label study of patients with chronic noncancer pain (Spierings et al., 2015). Lubiprostone administered at 24 μg twice daily for relief of OIC in patients with chronic noncancer pain does not interfere with opioid analgesia (Spierings et al., 2017).

Efficacy of lubiprostone on CIC, OIC, and IBS-C has been fully confirmed in clinical studies. In a randomized, double-blinded, placebo-controlled multicenter phase 3 trial, patients with OIC received lubiprostone (24 μg) twice daily for 12 weeks. The overall spontaneous bowel movements (SBM) were significantly increased and opioid-associated symptoms were significantly reduced (Jamal et al., 2015). In another randomized study (NCT00595946), patients orally received lubiprostone 24 μg twice daily for 12 weeks, OIC and associated symptoms were significantly relieved as compared to the placebo-treated patients (Cryer et al., 2014). In a phase 2 clinical study for IBS-C, patients received daily doses of 16, 32, or 48 μg lubiprostone for 3 months showed significant improvements in mean abdominal discomfort/pain scores (Johanson et al., 2008a).

Lubiprostone has little systemic absorption and has prompt onset of action. It was reported that bowel movement of patients with constipation was significantly improved within 24–48 h of initial dosing of lubiprostone (Johanson et al., 2008a,b). Lubiprostone is readily metabolized within the GI via microsomal carbonyl reductase with an estimated half-life of approximately 3 h. Lubiprostone's M3 active metabolite is well absorbed and is approximately 94% bound to plasma protein with a half-life of about 0.9–1.4 h. Animal studies using radiolabeled lubiprostone showed that it is almost completely cleared within 48 h (Soubra and Schey, 2012). Lubiprostone is generally well tolerated in patients; and the most common adverse events in lubiprostone treatment include nausea, diarrhea, headache, and vomiting (Li et al., 2016). However, there is one case report of dose-dependent lubiprostone-induced ischemic colitis in CIC patient (Sherid et al., 2013). Johanson and colleagues also reported dose-associated GI adverse events in lubiprostone-treated IBS-C patients (Johanson et al., 2008a). So far, lubiprostone has been approved by the US, UK, Japan and Switzerland for the treatment of CIC. The US FDA also approved its clinical use for the treatment of IBS-C and OIC in adults with chronic non-cancer pain in 2013 (Wilson and Schey, 2015). Lubiprostone was discovered by Dr Ryuji Ueno. There are only a few applications expanding on the preparation of lubiprostone and its intermediates (Qilu Pharmaceutical Co Ltd., 2015a,b; Scinopharm Kunshan Biochemical Technology Co Ltd., 2015).

### Linaclotide

Linaclotide (Linzess, Ironwood/Forest) (Figure 1C), a 14-amino acids peptide homologous to bacterial heat-stable enterotoxins, is a first-in-class anti-constipation drug targeting GC-C receptor on the luminal surface of GI enterocytes. Activation of GC-C receptor increases both intracellular and extracellular levels of cyclic guanosine monophosphate (cGMP). The increase in intracellular cGMP level triggers the activation of CFTR chloride channel activity and thereby results in salt and water secretion into the lumen of intestine. Linaclotide was also shown to attenuate nociceptive reflexes in rodent models mainly through increasing extracellular cGMP level (Eutamene et al., 2010).

Linaclotide was developed by Ironwood Pharmaceuticals. Efficacies of linaclotide have been fully confirmed in adult patients with constipation by various clinical trials. In a randomized, double-blinded, placebo-controlled, multicenter phase 2 study (NCT00402337), 310 patients with constipation randomized to series dosing of linaclotide (75, 150, 300, or 600 μg) or placebo once daily for 4 weeks. The results showed that linaclotide at all doses increased the overall weekly rate of SBM and the frequency of complete SBM (CSBM), decreased constipation-related abdominal symptoms such as straining, abdominal discomfort and bloating (Leyva-Vega et al., 2010). In three identical phase 3 randomized trials (NCT00765882, NCT00730015, NCT01642914), 1759 patients with constipation randomized to oral linaclotide (145 or 290 μg) or placebo once daily for 12 weeks, the data showed that linaclotide significantly improved bowel and abdominal symptoms in patients with moderate-to-severe abdominal bloating (Lembo et al., 2011; Lacy et al., 2015).

Linaclotide was also shown effective in patients with IBS-C. In a multicenter, double-blinded phase 2 study (NCT00460811), 420 patients with IBS-C were randomized to different doses (75, 150, 300, or 600 μg) of linaclotide or placebo. It showed a sustained improvement in frequency of SBM and CSBM that accompanied reduced straining and improved stool consistency in all dose of linaclotide groups as compared to placebo throughout 12 weeks of treatment (Johnston et al., 2010). In other multicenter, randomized, double-blinded, placebo-controlled phase 3 studies (NCT00938717, NCT00948818.), 1606 patients with IBS-C were randomized to linaclotide (290 μg) or matching placebo once daily for 12-weeks. The results showed that linaclotide significantly reduced abdominal pain in patients with severe symptoms and improved other bowel symptoms associated with IBS-C (Quigley et al., 2013; Rao et al., 2014). In 2012, the US FDA approved linaclotide for treatment of IBS-C and adults CIC. Shortly after, the drug was also approved by the European Medicines Agency (EMA) for the treatment of moderate to severe IBS-C in adults (McWilliams et al., 2010; Blackshaw and Brierley, 2013). The recommended dosage is 145 μg (for the treatment of IBS-C) or 290 μg (for constipation) orally once daily and 30 min prior to the first meal. The success of GC-C-targeted therapy of constipation greatly encourages the development of other emerging molecularly directed therapies for the disease, which includes the pro-kinetic serotoninergic 5-HT4 receptor agonist prucalopride and cGMP agonist plecanatide.

Like lubiprostone, linaclotide is also a luminally effective agent with only limited systemic absorption following oral administration. Linaclotide is resistant to protease and acid in the stomach and is readily converted to an active metabolite (MM-419447, amino acid sequence CCEYCCNPACTGC). After the reduction of their disulfide bonds, both linaclotide and MM-419447 will be proteolyzed and degraded into smaller peptides and amino acids in small intestine (Busby et al., 2013). Both linaclotide and MM-419447 are potent agonists of GC-C that can potently increase fluid secretion into small intestinal loops, accelerate GI transit and reduce visceral hypersensitivity (Busby et al., 2013). The most common adverse effect of linaclotide is mild to moderate diarrhea as expected from the drug's pharmacology. Due to the occurrence of death in juvenile mice, linaclotide is contraindicated in patients under 6 years of age and recommended to avoid using in patients 6–17 years old (Cada et al., 2013; Thomas and Allmond, 2013).

Ironwood Pharmaceuticals Inc. claimed the use of linaclotide for the treatment of constipation and other GI disorders in 3 files (Ironwood Pharmaceuticals Inc., 2011a,b, 2013). Linaclotide may be prepared through solid-phase synthesis, solution phase synthesis, sequential adding peptide and fragment based coupling methods, among which solid-phase synthesis is so far the most promising method. Methods for linaclotide synthesis are the most competitive field of patent application. Recently, several applications claimed the preparation, stable improvement and purification methods of linaclotide (Lonza, 2014; Auro Peptides Ltd., 2015, 2016). Methods of linaclotide preparation in these applications are similar, all through solid-phase synthesis method by using automated peptide synthesizers. Differences among these methods mainly include solvent, coupling and decoupling methods and oxidation methods used in the process. LONZA AG [CH] claimed a solid phase peptide synthesis method for the preparation of linaclotide (Lonza, 2014). They also provided deprotection, oxidation and purification methods in the application. Pool purity of the final product is more than 97% as indicated by reverse phase high performance liquid chromatography (RP-HPLC), while no yield data is provided in the application. Two applications expand on the preparation of linaclotide were claimed by AURO PEPTIDES LTD (Auro Peptides Ltd., 2015, 2016). The former application provides a solid phase peptide synthesis method with an oxidized purity around 70%. After purified by RP-HPLC method, purity of linaclotide was increased to >98.5% (Auro Peptides Ltd., 2015). By optimizing the synthesis methods, AURO PEPTIDES LTD increased the oxidized purity and final product purity to 74 and 98.9% (Auro Peptides Ltd., 2016), respectively. REDDY'S LAB LTD discloses methods related to improved processes for the preparation of amorphous linaclotide, the formation of disulfide bonds in linaclotide as well as purification methods. They disclosed preparation of linaclotide in different solvents and different oxidation conditions. The data showed that final purity of the product reaches 99% (Reddy's Lab Ltd., 2016).

Stability of linaclotide dosage forms has presented a significant problem to the formula. Linaclotide possesses intrinsic chemical instability including moisture-driven degradation reactions such as hydrolysis, oxidation, deamidation, isomerization, and multimerization (Ironwood Pharmaceuticals Inc., 2015). It is necessary to provide novel methods of preparing a stable solid dosage form of linaclotide with improved shelf life and robust stability profiles. Two applications claimed methods for stable pharmaceutical compositions that comprise linaclotide and pharmaceutical acceptable salts. Forest Labs/Ironwood (Ironwood Pharmaceuticals Inc., 2015) claimed a stable pharmaceutical composition comprising linaclotide, Ca^2+^ and histidine. After the compositions stored at stressed conditions (40°C and 75% humidity) for up to 18 months, the amount of linaclotide remains no significant change. SUN PHARMACEUTICAL IND LTD also disclosed methods of stabilizing linaclotide in a solid dosage form, in which linaclotide, acesulfame and pharmaceutical acceptable excipients were mixed and converted into solid dosage forms. After stored at 25°C and 60% relative humidity condition for 3 months, no significant change in linaclotide content was found in the solid dosage (Sun Pharmaceutical Ind Ltd., 2016).

### Plecanatide

Plecanatide (SP-306) (Figure 1D), a 16 amino acid GC-C activator, is structurally identical to uroguanylin except for an amino acid difference on N-terminus. Similar to linaclotide, plecanatide also activates GC-C receptors located on the luminal surface of intestinal enterocytes, leading to the elevation of intracellular cGMP and subsequent activation of CFTR chloride channel (Jiang et al., 2015). Systemic absorption of plecanatide is very low. According to a phase 1 study done on 72 healthy volunteers, oral administration of a single dose of plecanatide up to 48.6 mg was verified safe and well-tolerated (Shailubhai et al., 2013).

Plecanatide is the second synthetic GC-C agonist, which is currently under development for the treatment of CIC and IBS-C by Synergy Pharmaceuticals Inc. In a Phase 2b/3, randomized, double-blind, placebo-controlled study (NCT01429987) in 951 adult patients aged 18–75 with CIC orally dosing plecanatide (0.3, 1.0, and 3.0 mg) or placebo once daily for 12 weeks, the results indicated that significant reduction in constipation-related symptoms were occurred in 3 mg dose group compared to the placebo (Thomas and Luthin, 2015). Efficacy of plecanatide was also evaluated in two identical phase 3 studies (NCT02122471, NCT01982240). However, no official data were released from these clinical trials so far. Another randomized, 12-week, double-blind, placebo-controlled, dose-ranging (0.3, 1.0, and 3.0 mg) phase 2 study in 350 patients age 18–75 with IBS-C was completed in January 2015 (NCT01722318). No result is released from the study either. Currently, two randomized, double-blind, placebo-controlled, dose-ranging (3, 6 mg), 12-week, phases 3 clinical trials (NCT02387359 and NCT02493452) are recruiting participants, each study will include 1,050 patients. A phase 3, multicenter, open-label, long-term safety and tolerability study of 6 mg daily dose of plecanatide administered orally is currently underway (NCT02706483). Recently, plecanatide was approved in the USA for the treatment of CIC in adult patients and a phase III investigation in IBS-C in undergoing (Al-Salama and Syed, 2017).

There are three applications from Nanjing University of Technology, China expanding on methods for preparation of plecanatide (Shenzhen Hybio Pharmaceutical, 2013a,b; Univ Nanjing Tech, 2015). The data showed that the yielding rate and purity of the final product could be up to 37.88 and 99.47%, respectively, in certain specified optimized solid phase peptide synthesis routes.

### Elobixibat

Elobixibat (A3309) (Figure 1B), a first-in-class ileal bile acid transporter (IBAT) inhibitor, is now developed by Ferring Pharmaceuticals, Mayo Clinic and Albireo, respectively. Elobixibat accelerates colonic transit through enhancing delivery of bile acids to the colon, and thus increasing stool frequency as well as decreasing constipation-related symptoms in CIC patients (Acosta and Camilleri, 2014). Efficacy of elobixibat in CIC treatment has been confirmed by several clinical trials sponsored by Albireo. In a single-center, double-blind, placebo-controlled phase 2 study (NCT01038687), 36 patients with CIC were randomized to elobixibat oral tablets (15 or 20 mg once daily) or placebo for 2 weeks. It showed that eloxibibat at both dosages significantly accelerated colonic transit and loosened stool consistency in the patients (Wong et al., 2010) In a multi-center phase 2b study, 190 adult CIC patients were randomized to 5, 10, or 15 mg eloxibibat or placebo once daily for 8 weeks (NCT01007123). The results showed that the time to the first SBM and CSBM were significantly shortened in the 10- and 15-mg groups, and stool frequency and constipation-related symptoms were significantly improved (Chey et al., 2011).

## ORL-1 receptor antagonists

Opioids are the most commonly used analgesics for severe and non-malignant pain. Although opioids are effective in alleviating severe pain, they cause numerous adverse effects such as physical dependence, sedation and constipation that undermine their clinical utilities. OIC is difficult to manage and significantly reduces quality of life in opioid users. Mechanisms underlying OIC arise from opioid-mediated actions on not only the central nervous system (CNS) but also on GI tract (Günther et al., 2017). In GI tract, opioids mainly agonize μ receptor and opioid receptor-like-1 (ORL-1) in the enteric system to reduce GI propulsion, which prolongs intestinal transit time (Nelson and Camilleri, 2016).

**Figure 2:**
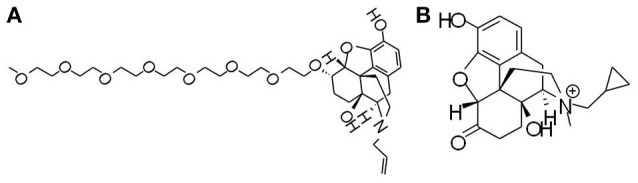
Chemical structures of opioid receptor antagonists. **(A)** Naloxegol, a polyethylene glycol derivative (PEGylated) of naloxone, is the first approved μ-opioid receptor antagonist (PAMORA) for the treatment of opioid-induced constipation (OIC) in non-cancer patients. **(B)** Methylnaltrexone bromide, also targeting PAMORA, decreases OIC symptoms without compromising central analgesia.

Currently, approved ORL-1 receptor antagonists available for OIC treatment include naloxegol (Figure 2A), naloxone (an oral combination of oxycodone and naloxone) and methylnaltrexone (Figure 2B). Naloxegol (developed by AstraZeneca), a polyethylene glycol derivative (PEGylated) of naloxone, is the first orally available, peripherally acting, μ-opioid receptor antagonist (PAMORA) approved by the US FDA and EMA specifically for the treatment of OIC in non-cancer patients (Burness and Keating, 2014; Tack and Corsetti, 2014a; Corsetti and Tack, 2015a,b; Leonard and Baker, 2015). Naloxegol reduces OIC through antagonizing the μ receptor in the GI tract without reducing its central analgesic effect (Chey et al., 2014). Efficacy and safety of naloxegol have been fully confirmed by numerous clinical tests. In two identical, multicenter, randomized, double-blind, placebo-controlled phase 3 studies (NCT01309841, NCT01323790), patients aged 18–84 with non-cancer-related pain and OIC were assigned to receive naloxegol (12.5 or 25 mg) oral tablet once daily for 12 weeks. Significant response including the median time to the first SBM, mean number of SBMs/week, number of SBM/week, severity of straining, stool consistency, and frequency of days with CSBM was achieved in both dosage groups as compared to the placebo. The most reported adverse effects related to naloxegol are GI symptom like abdominal pain, diarrhea, nausea and vomiting occurring in 25 mg group. No serious adverse event was observed in the clinical trials (Chey et al., 2014). Phase 3 study of naloxegol on refractory constipation in the intensive care unit and phase 2 study of naloxegol in cancer OIC are under way. Because naloxegol is primarily cleared by the hepatic route and hepatically impaired patients exhibited a 17–18% decrease in area under the plasma concentration-time curve (AUC), it is suggested that naloxegol should be cautiously used in patients with moderate and severe renal impairment (Bui et al., 2014).

Methylnaltrexone bromide, also a PAMORA, was approved 12 mg subcutaneous injection every other day by the US FDA and the EMA for the treatment of OIC in patients with advanced illness in 2008. Methylnaltrexone bromide is a luminal active agent that decreases OIC symptoms without compromising centrally mediated analgesia (Guay, 2009). According to results from randomized clinical trials published in recent years, safety and efficacy of methynaltrexone seems not being fully confirmed yet. Details is seen in review articles (Mehta et al., 2016; Siemens and Becker, 2016). Naloxone is a combination of prolonged release (PR) xycodone with PR naloxone, an orally effective formulation that has been approved by the US FDA to counteract OIC (Nelson and Camilleri, 2016). Other PAMORA drugs are now under development for OIC include axelopran, naldemedine, alvimopan. Details can be seen in a review article (Nelson and Camilleri, 2016).

Purdue Pharma L.P. is in the leading place in the development of morphine analogs as opioid and ORL-1 receptor modulators. They claimed a series of benzomorphan and buprenorphine analogs in at least 7 filings to expand worldwide rights on structure and synthesis methods as opioid and/or ORL-1 receptor modulators for the treatment of OIC (Purdue Pharma Lp, 2012, 2013a,b, 2014a,b,c, 2015). The most effective compound has an EC_50_ value less than 1 nM, and certain of the applied compounds showed similar efficacy to that of the standard μ agonist (DAMGO) in prevention of pain. No anti-constipation data of these compounds are available yet from these applications. Adolor Corporation disclosed 5-(2-methoxy-4-{[2-(tetrahydro-pyran-4-yl)-ethylamino]-methyl}-phenoxy)-pyrazine-2-carboxamide as an opioid receptor antagonists (Adolor Corporation, 2011). The compound has high affinity to μ opioid receptor with a Ki value of 0.36 nM. The lowest intravenous dose of the applied compound to produce antagonism of opioid receptors in the CNS (around 3–10 mg/kg) is much higher than that required to reverse GI transit inhibition in mice (0.16 mg/kg), suggesting that the compound can be used as an anti-constipation agent for treatment of OIC without attenuating the analgesic effect of opioids.

## Prokinetic agents (5-HT4 receptor agonists)

Enhancing intestinal motility is an important way for constipation therapy. The activities of GI tract, such as motility, epithelial secretion and vasodilation, are coordinately regulated by enteric nervous system (ENS) for digestion and defecation. Serotonin (5-HT), released from the enterochromaffin cells in the intestinal mucosa, is one of the most abundant molecules in the GI tract. As an important signaling molecule in the gut, 5-HT is closely associated with many physiological functions (like GI motility, secretion, and sensation) and pathological process (like immune/inflammatory responses), although the precise functions are confusing and controversial. It has been well defined that mucosal 5-HT signaling changes are associated with both IBS-C and IBS-D, but how a shared defects in 5-HT signaling lead to such distinct pathophysiological symptoms as diarrhea and constipation in IBS remains undetermined (Coates et al., 2004). Several lines of evidence indicated that mucosal 5-HT signaling is altered in CIC with different patterns from those found in IBS-C or IBS-D. In IBS-C and IBS-D, decreased content of mucosal 5-HT and expression of serotonin-selective reuptake transporter (SERT) were detected (Coates et al., 2004; Camilleri et al., 2007a). However, in CIC the altered 5-HT signaling seems to result from increased 5-HT availability rather than altered SERT expression (Camilleri et al., 2007b; Costedio et al., 2010).

**Figure 3:**
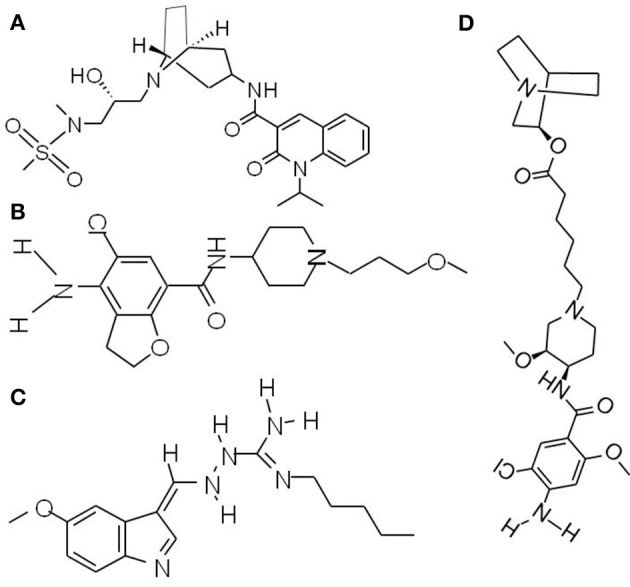
Chemical structures of prokinetic agents (5-HT4 receptor agonists). **(A)** Velusetrag, a 5-HT4 receptor agonists, significantly increases intestinal and colonic transit. **(B)** Prucalopride, a first-in-class dihydro-benzofurancarboxamide derivative, is a highly selective agonist of 5-HT4 receptor. **(C)** Tegaserod, the first 5-HT4 receptor agonist for short-term IBS-C treatment in women. **(D)** Naronapride, a highly selective 5-HT4 receptor agonist, significantly accelerates overall colonic transit.

The wide range of pathophysiological actions of 5-HT are accomplished via different receptor types and subtypes. In gut, 5-HT mediates prosecretory effects mainly through interacting with 5-HT3 and 5-HT4 receptors, which have been validated as molecular targets for the treatment of GI motility disorders (Gershon and Tack, 2007). So far, a number of 5-HT4 receptor agonists, including velusetrag (TD-5108) (Figure 3A), prucalopride (Figure 3B) and tegaserod (Figure 3C), naronapride (ATI-7505) (Figure 3D), have been under clinical investigation or approved for constipation therapy.

Tegaserod (Zelnorm, Novartis) was approved by US FDA in 2002 for short-term IBS-C treatment in women and in 2004 for CIC therapy in adult patients. Tegaserod increases peristaltic activity and drives fluid secretion in the intestine, thus facilitating stool passage. It also helps reduce IBS-C related abdominal pain through modulating visceral sensitivity. Efficacy of tegaserod in IBS-C and CIC therapies have been fully confirmed by several clinical investigations (Tougas et al., 2002; Quigley et al., 2006; Fock and Wagner, 2007). Generally, tegaserod is well tolerated and associated with few drug interactions. The side effects of the drug may include diarrhea, abdominal pain, flatulence, headache and nausea. However, because a relatively high rate of cardiac ischemic events occurred in patients with cardiovascular risk factors was reported, use of tegaserod has been restricted only in emergency situation by the US FDA in 2007. Safety and efficacy evaluations of tegaserod in patients with non-cancer pain and OIC (NCT00399659) were terminated as the result of regulatory action suspending tegaserod use in 2007.

Prucalopride, a first-in-class dihydro-benzofurancarboxamide derivative, is a highly selective agonist of 5-HT4 receptors. Safety and efficacy of prucalopride has been confirmed by a series of clinical studies. In three identical multicenter, double-blind, placebo-controlled phase 3 studies [NCT00485940, NCT00483886, NCT00488137], 1977 patients with chronic constipation were assigned to prucalopride (2 or 4 mg) or placebo once daily for 12 weeks. The results showed that the severity of constipation-related symptoms, including abdominal pain, abdominal discomfort, bloating, cramps, straining and painful bowel movements, was significantly alleviated in both 2 mg and 4 mg prucalopride groups and there was no additional benefit with the 4 mg over the 2 mg dosage (Tack et al., 2009, 2014b, 2015). Prucalopride has been approved in the European Union at a recommended dose of 2 mg/day for the treatment of CIC in women who failed to respond to laxatives (Tack et al., 2014b). In comparison to tegaserod, prucalopride manifests more favorable safety and tolerability profiles even in elderly subjects with stable cardiovascular diseases, which may be attributed to its higher affinity with 5-HT4 receptor. Several clinical studies showed that prucalopride at dosages up to 10 mg per day (5-fold higher than the recommended therapeutic dosage) had no clinically relevant effects on cardiovascular parameters in healthy volunteers (NCT00793429, NCT01870674). Efficacy and safety data of prucalopride have been systematically reviewed by Sajid and colleagues (Goldberg et al., 2010).

Theravance Biopharma R & D, Inc. sponsored a phase 2 clinical trial to evaluate the efficacy and tolerability of velusetrag (TD-5108) in patients with CIC (NCT00391820), in which 360 patients aged 18–64 with CIC were received placebo or velusetrag (15, 30, or 50 mg) once daily for 4 weeks. The results indicated that patients receiving velusetrag at all dosages achieved clinically significant improvement in weekly SEM as compared to the placebo group: 3.3–36 SEM/week (velusetrag-treated group) vs 1.4 SEM/week (placebo group) (Sajid et al., 2016). In a clinical study approved by the Mayo Clinic Institutional Review Board, 60 health volunteers (aged 18–65) were randomly assigned to 1 of 4 velusetrag doses (5, 15, 30, and 50 mg, single and 6-day dosing) or placebo. The results showed significantly increased intestinal and colonic transit after single dosing, and gastric emptying was accelerated after multiple dosing (Manini et al., 2010). A phase 2 study to assess the safety and tolerability of velusetrag in diabetic or idiopathic gastroparesis have been completed (NCT01718938), but data was not available so far.

Naronapride (ATI-7505) is a highly selective 5-HT4 receptor agonist that has little action on the other 5-HT receptor subtypes. Naronapride has a plasma terminal half-life around 5.36 h and is eliminated mainly via fecal excretion (Bowersox et al., 2011). Naronapride (10 mg t.i.d.) significantly accelerated overall colonic transit and tended to accelerate gastric and colonic emptying in healthy human (Camilleri et al., 2007b). A Phase 2, randomized, placebo-controlled study of naronapride (ATI-7505) in patients with CIC sponsored by Procter and Gamble has been completed (NCT00501241), but up to now no result was released.

## Conclusion

**Figure 4:**
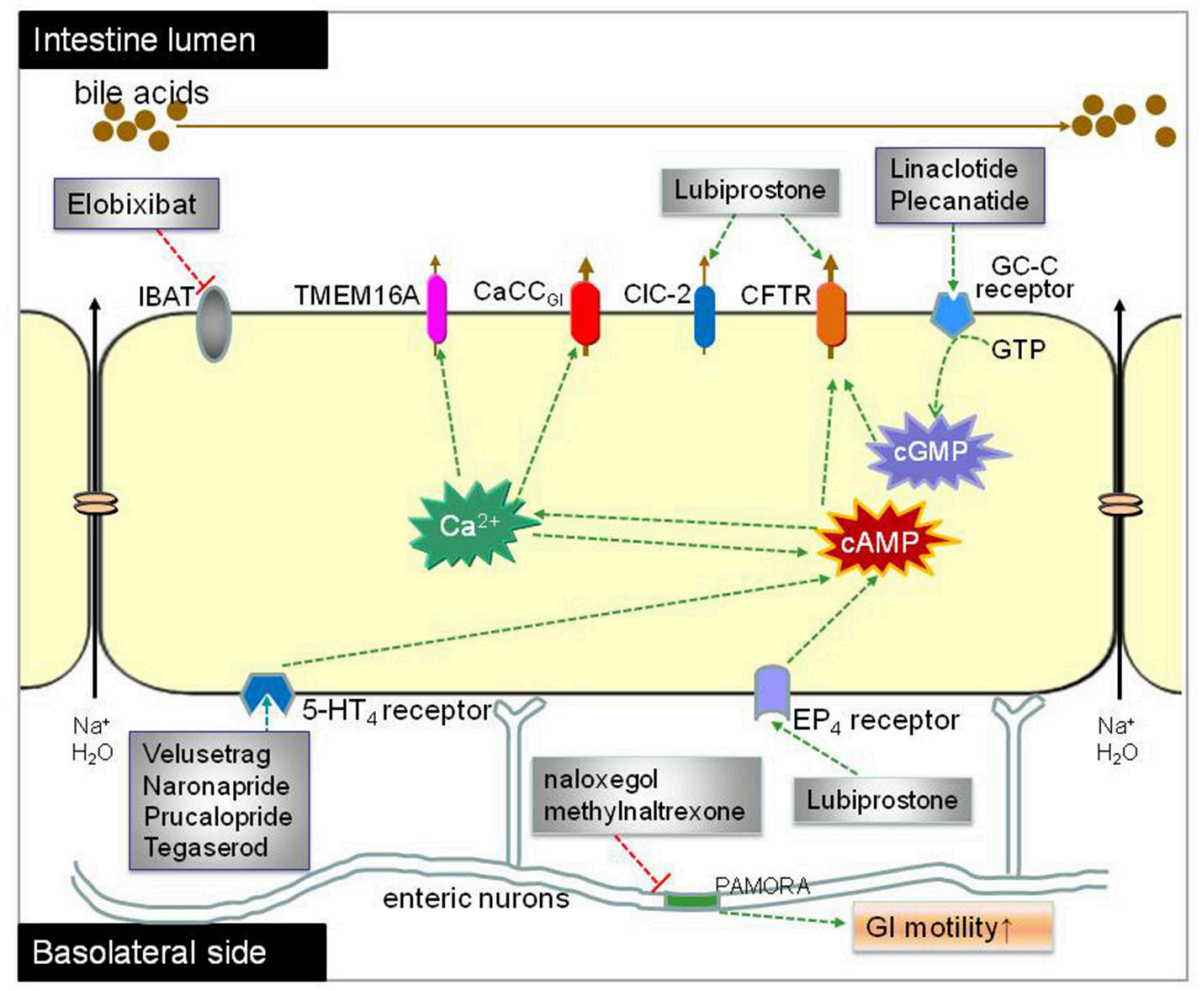
Proposed mechanisms of luminally acting agents for constipation treatment. The prosecretory agents linaclotide and plecanatide activate CFTR chloride channel in the enterocytes by elevating intracellular cGMP level through GC-C receptor and thereby enhancing salt and water secretion. Lubiprostone stimulates CFTR-dependent chloride secretion directly and/or via EP4 receptor pathway. Elobixibat accelerates colonic transit through enhancing delivery of bile acids to the colon, thus increasing stool frequency in CIC patients. The ORL-1 receptor antagonists naloxegol and methylnaltrexone reduce OIC through antagonizing the μ-receptor in the GI tract without reducing its central analgesic effect. Velusetrag, naronapride, prucalopride and tegaserod are selective agonists of 5-HT4 receptor and increase transepithelial secretion of Cl^−^ and ${\text{HCO}}_{3}^{-}$.

Table 1 summarizes the common doses used in treatment and the common side effects. Increasing stool water content with prosecretory agents and enhancing intestinal motility with prokinetic agents compromise two main strategies for anti-constipation therapy. Success of prosecretory agents like lubiprostone and linaclotide in anti-constipation therapy has proven conclusively the feasibility of chloride channel-targeted therapy in constipation treatment. CFTR-dependent chloride secretion is the common pathway for most of the proved (lubiprostone and linaclotide) and emerging (plecanatide) prosecretory agents, although molecular mechanisms of these agents may be different. Although common adverse events such as mild to moderate diarrhea in prosecretory treatment usually are regarded as a matter of course from the drug's pharmacology, the occurrence of death caused by linaclotide in animal studies highlights the necessity for further systematic investigation of pharmacological functions of these agents. Limited efficacy and expensiveness of linaclotide and lubiprostone urges more potent chemicals for anti-constipation therapy. Prokinetic agents aimed at enhancing intestinal motility is also approved an alternative way for anti-constipation therapy. Stimulating 5-HT4 signaling is the main strategy for the approved (tegaserod and prucalopride) and promising (velusetrag and naronapride) prokinetic agents. Despite the verified efficiency in anti-constipation therapy, tegaserod confronts challenges due to a relatively high rate of cardiac ischemic events in patients with cardiovascular risk factors. Prokinetic agents (like naronapride) with more selectivity and high affinity to 5-HT4 receptor have proved more favorable safety and tolerability profiles in elderly subjects with stable cardiovascular diseases. Currently available medications for OIC therapy, including naloxegol, naloxone and methylnaltrexone, target at PAMOR. An ideal anti-constipation agent should antagonize the μ receptor in the GI tract without compromising opioid-mediated analgesia actions in the CNS. Figure 4 summerizes molecular targets of current luminally active anticonstipation agents.

**Table 1:** Luminally acting agents for constipation treatment.

**Generic name (brand name)**	**Mechanism of action**	**Indication**	**Common dosages**	**Status**	**Common adverse**
**PROSECRETORY AGENTS**					
Lubiprostone (Amitiza)	ClC-2 and CFTR activator, EP4 receptor agonist	CIC, OIC, IBS-C	24 μg b.d.oral	Approved in the US (CIC, OIC and IBS-C), UK (CIC), Japan (CIC) and Switzerland (CIC)	Nausea, diarrhea, headache, vomiting
Linaclotide (Linzess)	GC-C receptor agonist	CIC, IBS-C	145 μg (IBS-C) or 290 μg (CIC) daily oral	Approved in the US (IBS-C and adults CIC) and EMA (IBS-C in adults)	Diarrhea
Plecanatide (TrulanceTM)	GC-C receptor agonist	CIC, IBS-C	6 mg daily oral	Approved in the US (CIC), Phase 3 (IBS-C)	
Elobixibat (A3309)	Partial inhibition of ileal bile acid transporter	CIC	15 mg daily oral	Phase 3 (CIC)	Abdominal pain and diarrhea
**OPIOID AGONISTS AND ORL-1 RECEPTOR ANTAGONISTS**					
Naloxegol	μ-opioid receptor antagonist	OIC	15 mg daily oral	Approved in the US and EMA	Abdominal pain, diarrhea, nausea and vomiting
Methylnaltrexone	μ-opioid receptor antagonist	OIC	12 mg subcutaneous injection every other day	Approved in the US and EMA	Abdominal pain, nausea, and diarrhea
**5-HT4 AGONIST**					
Velusetrag (TD-5108)	Highly selective 5-HT4 receptor agonist	CIC	15 mg daily oral	Phase 2 (limited data available)	
Naronapride (ATI-7505)	Highly selective 5-HT4 receptor agonist	GI motility disorders	10 mg t.i.d. oral	Phase 2 study (limited data available)	
Prucalopride (RO93877)	Highly selective 5-HT4 receptor agonist	CIC	2 mg daily oral	Approved in EMA (CIC)	Diarrhea, headache
Tegaserod (Zelnorm)	Nonselective 5-HT4 receptor agonists	CIC, IBS-C	2 m or 6 mg b.d. oral	Limited to emergency use in the US	Diarrhea, abdominal pain, flatulence, headache and nausea

## Perspective

Appropriate stool water content and intestinal motility are essential for normal defecation. Intestinal fluid secretion is mainly through Cl^−^ secretion across the enterocyte epithelium, which creates electrochemical and osmotic forces to drive Na^+^ and water secretion. Therefore, chloride channels on the luminal surface of enterocytes play critical roles in intestinal fluid secretion and may become ideal targets for constipation therapy. CFTR and CaCC chloride channels are robustly expressed throughout the intestine and comprise the common pathways for active Cl^−^ secretion across the intestinal epithelium.

Because of the massive intestinal fluid secretion mediated by activation of the CFTR in cholera and enterocyte CaCC in rotavirus infections (Ko et al., 2014), we postulated that CFTR and enterocyte CaCC activators would increase intestinal fluid secretion and treat constipation. In a recent proof-of-concept study, it was found that a small-molecule CFTR-targeted activator (CFTRact-J027) is efficacious for the treatment of constipation (Cil et al., 2016, 2017). Such treatment does not produce a global cyclic nucleotide response in multiple cell types, which merits its potential use as promising anti-constipation agent (Cil et al., 2016). Limited efficacy and expensiveness of linaclotide and lubiprostone urges more potent chemicals for the treatment of constipation. One of the future challenges is to integrate prosecretory agents with bulk water movement and to co-administrate agents targeting several molecular targets to achieve synergistic effects.

Enhancing intestinal motility provides another choice for constipation therapy. Commonly used drugs for the purpose of stimulation of GI smooth muscle contraction include diphenylmethanes, anthraquinones, misoprostol, and castor oil. Mechanisms of actions of these drugs has not been well defined, which may include stimulating sensory nerves on colonic mucosa, irritating colonic wall, inhibiting water absorption as well as increasing prostaglandin production. The most common adverse effects of these stimulant agents include bloating, nausea, diarrhea and even damages of enteric nerves and smooth muscles.

**Figure 5:**
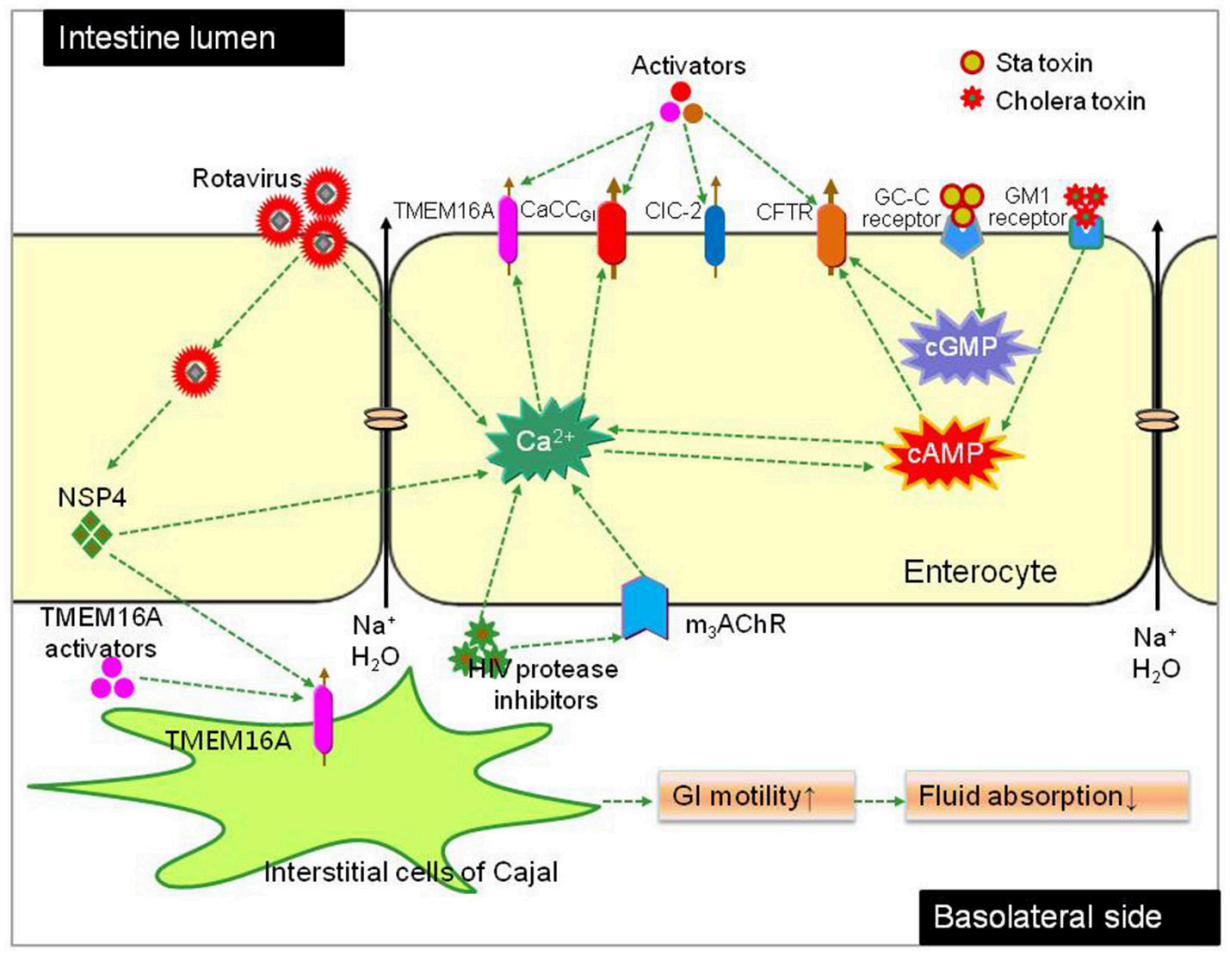
Chloride channels as molecule targets for constipation therapy. Fluid secretion across the intestinal epithelia is mainly driven by Cl^−^ transport through chloride channels located in apical membrane of enterocytes. CFTR and a CaCC (CaCC_GI_, molecular identity unknown) have been found to be the major chloride channels in bacterial and viral diarrhea respectively. Bacteria toxin (like cholera and Sta toxin) increase intracellular cAMP, resulting in CFTR-mediated Cl^−^ secretion. The rotaviral protein NSP4 and HIV protease inhibitors cause elevation of cytoplasmic Ca^2+^ concentration, resulting in CaCC_GI_-mediated Cl^−^ secretion; NSP4 also activates TMEM16A in the membrane of interstitial cells of Cajal (ICCs), resulting enhanced GI motility. Chloride channel activators targeting different chloride channels can stimulate fluid secretion into the lumen or increase intestinal motility.

GI motility is a complex process involving coordination and communication of multiple cell types including enteric neurons, ICCs, and smooth muscle cells. ICCs, specialized cells found throughout the GI tract from the esophagus to the internal anal sphincter (Sanders and Ward, 2006), are the pacemaker cells in GI smooth muscles. ICCs play important roles in GI motility such as generation of electrical slow waves and mechano-transduction, participation of cholinergic and nitrergic neurotransmission, and setting of the smooth muscle membrane potential (Klein et al., 2013). ICCs have been implicated in the pathogenesis of many GI disorders including diabetic gastroenteropathy, Hirschsprung's disease, chronic idiopathic intestinal pseudo-obstruction, anorectal malformations, infantile hypertrophic pyloric stenosis, achalasia and gastro-esophageal reflux disease (He et al., 2001; Bettolli et al., 2008; Hwang et al., 2009), although the mechanisms underlying these functions remain largely unveiled. Recent studies indicated that TMEM16A is involved in GI pacemaker activity. Contractility of intestinal segments of TMEM16A knockout mouse was significantly decreased, less coordinated and non-rhythmic (Singh et al., 2014). In 2011, Verkman group found that the TMEM16A-selective inhibitor T16A_inh_-01 potently reduced smooth muscle contraction in mouse intestine *ex vivo*, while the TMEM16A activator E_act_ increased intestinal motility and restored contraction following atropine inhibition (Namkung et al., 2011; Yao et al., 2012). These data suggest that GI smooth muscle motility can be modulated by pharmacological activation or inhibition of TMEM16A channel activity. Therefore, it is reasonable to presume that TMEM16A activators may be of therapeutic utility for GI motility disorders such as STC, and inhibitors for disorders associated with hypermotility. Figure 5 summerizes chloride channels as potential drug targets for constipation therapy.

Recognization of the role of CFTR and CaCC chloride channels in intestinal fluid secretion and motility modulation makes them promising molecular targets for constipation therapy. To date, numerous CFTR activators have been identified and widely used in investigation of CFTR pathophysiological functions and even represented clinical options for anti-constipation therapy (like lubiprostone and linaclotide). The CaCC activators discovered so far include E_act_ and F_act_ identified by Verkman group in 2011 from combinatorial small molecule library. In *ex vivo* studies, both E_act_ and F_act_ could significantly stimulate smooth muscle contraction in mouse intestine without elevating cytoplasmic Ca^2+^ level (Namkung et al., 2011). The relatively low hit rate of CaCC activators urges to establish new compound libraries with high structural diversities. Natural compounds library possess the virtue of abundant chemical structures. However, it is hard to generate at large scale, which hammered the interest of pharmaceutical industry to develop. A recently reported natural product fraction library may be useful for this purpose, in which traditional Chinese herbal medicines were extracted and fractionated into simple fractions containing a small number of compounds. The feasibility of the fraction library was verified by the F508del-CFTR corrector screening; and the unexpectedly high “hit” rate of the fraction library suggests its potential use for low hit rate (like CaCC activators and inhibitors) screening.

Although functions of CaCCs have been confirmed, the only CaCCs characterized include TMEM16A and TMEM16B (ANO2). TMEM16B is not expressed in enterocytes, while TMEM16A contribute only a minor part to intestinal Cl^−^ conductance. Therefore, it is critical to unveil the molecular identity of enterocyte CaCC. Small molecule activators are not only useful for drug therapy of constipation but also for pharmacological dissection of different types of CaCCs. In addition, because of the ubiquitous expression and multifunctions of CaCC and CFTR, it is povital to find activators that are orally effective with minimal systemic absorption.

## Author contributions

All authors listed have made a substantial, direct and intellectual contribution to the work, and approved it for publication.

### Conflict of interest statement

The authors declare that the research was conducted in the absence of any commercial or financial relationships that could be construed as a potential conflict of interest.

## References

[B1] Acosta A. Camilleri M. (2014). Elobixibat and its potential role in chronic idiopathic constipation. Therap. Adv. Gastroenterol. 167–175. 10.1177/1756283X14528269 25057297 PMC4107709

[B2] Adolor Corporation (2011). Use of Opioid Receptor Antagonist for Gastrointestinal Tract Disorders. WO035142(A1). Exton: Adolor Corporation.

[B3] Akiba Y. Kaunitz J. D. (2012). May the truth be with you: lubiprostone as EP receptor agonist/ClC-2 internalizing “inhibitor.” Dig. Dis. Sci. 57, 2740–2742. 10.1007/s10620-012-2410-2 23001408

[B4] Al-Salama Z. T. Syed Y. Y. (2017). Plecanatide: first global approval. Drugs 77, 593–598. 10.1007/s40265-017-0718-0 28255961

[B5] Altomare D. F. Portincasa P. Rinaldi M. Di Ciaula A. Martinelli E. Amoruso A.. (1999). Slow-transit constipation: solitary symptom of a systemic gastrointestinal disease. Dis. Colon. Rectum. 42, 231–240. 10211501 10.1007/BF02237134

[B6] Andromanakos N. P. Pinis S. I. Kostakis A. I. (2015). Chronic severe constipation: current pathophysiological aspects, new diagnostic approaches, and therapeutic options. Eur. J. Gastroenterol. Hepatol. 27, 204–214. 10.1097/MEG.0000000000000288 25629565

[B7] Ashraf W. Park F. Lof J. Quigley E. M. (1996). An examination of the reliability of reported stool frequency in the diagnosis of idiopathic constipation. Am. J. Gastroenterol. 91, 26–32. 8561138

[B8] Auro Peptides Ltd (2015). A Process for the Preparation of GC-C Agonist. WO022575 (A3). Hyderabad: Auro Peptides Ltd.

[B9] Auro Peptides Ltd (2016). A Process for the Preparation of Linaclotide. WO038497 (A1). Hyderabad: Auro Peptides Ltd.

[B10] Bassotti G. Stanghellini V. Chiarioni G. Germani U. De Giorgio R. Vantini I. (1996). Upper gastrointestinal motor activity in patients with slow-transit constipation. Further evidence for an enteric neuropathy. Dig. Dis. Sci. 141, 1999–2005. 10.1007/BF02093603 8888714

[B11] Bettolli M. De Carli C. Jolin-Dahel K. Bailey K. Khan H. F. Sweeney B.. (2008). Colonic dysmotility in postsurgical patients with Hirschsprung's disease. Potential significance of abnormalities in the interstitial cells of Cajal and the enteric nervous system. J. Pediatr. Surg. 43, 1433–1438. 10.1016/j.jpedsurg.2007.10.067 18675631

[B12] Blackshaw L. A. Brierley S. M. (2013). Emerging receptor target in the pharmacotherapy of irritable bowel syndrome with constipation. Expert Rev. Gastroenterol. Hepatol. 7, 15–19. 10.1586/17474124.2013.820045 23859756

[B13] Bowersox S. S. Lightning L. K. Rao S. Palme M. Ellis D. Coleman R. (2011). Metabolism and pharmacokinetics of naronapride (ATI-7505), a serotonin 5-HT(4) receptor agonist for gastrointestinal motility disorders. Drug. Metab. Dispos. 239, 1170–1180. 10.1124/dmd.110.037564 21447732

[B14] Bui K. She F. Sostek M. (2014). The effects of mild or moderate hepatic impairment on the pharmacokinetics, safety, and tolerability of naloxegol. J. Clin. Pharmacol. 54, 1368–11374. 10.1002/jcph.348 24945932

[B15] Burness C. B. Keating G. M. (2014). Oxycodone/Naloxone prolonged-release: a review of its use in the management of chronic pain while counteracting opioid-induced constipation. Drugs 74, 353–375. 10.1007/s40265-014-0177-9 24452879

[B16] Busby R. W. Bryant A. P. Bartolini W. P. Cordero E. A. Hannig G. Kessler M. M.. (2010). Linaclotide, through activation of guanylate cyclase C, acts locally in the gastrointestinal tract to elicit enhanced intestinal secretion and transit. Eur. J. Pharmacol. 649, 328–335. 10.1016/j.ejphar.2010.09.019 20863829

[B17] Busby R. W. Kessler M. M. Bartolini W. P. Bryant A. P. Hannig G. Higgins C. S.. (2013). Pharmacologic properties, metabolism, and disposition of linaclotide, a novel therapeutic peptide approved for the treatment of irritable bowel syndrome with constipation and chronic idiopathic constipation. J. Pharmacol. Exp. Ther. 344, 196–206. 10.1124/jpet.112.199430 23090647

[B18] Cada D. J. Levien T. L. Baker D. E. (2013). Linaclotide. Hosp. Pharm. 48, 143–152. 10.1310/hpj4802-143.test 24421452 PMC3839478

[B19] Camilleri M. (2015). Guanylate cyclase C agonists: emerging gastrointestinal therapies and actions. Gastroenterology 148, 483–487. 10.1053/j.gastro.2015.01.003 25576859

[B20] Camilleri M. Andrews C. N. Bharucha A. E. Carlson P. J. Ferber I. Stephens D.. (2007a). Alterations in expression of p11 and SERT in mucosal biopsy specimens of patients with irritable bowel syndrome. Gastroenterology 132, 17–25. 10.1053/j.gastro.2006.11.020 17241856 PMC2474784

[B21] Camilleri M. Vazquez-Roque M. I. Burton D. Ford T. McKinzie S. Zinsmeister A. R.. (2007b). Pharmacodynamic effects of a novel prokinetic 5-HT receptor agonist, ATI-7505, in humans. Neurogastroenterol. Motil. 19, 30–38. 10.1111/j.1365-2982.2006.00865.x 17187586

[B22] Castro J. Harrington A. M. Hughes P. A. Martin C. M. Ge P. Shea C. M.. (2013). Linaclotide inhibits colonic nociceptors and relieves abdominal pain via guanylate cyclase-C and extracellular cyclic guanosine 3′,5′-monophosphate. Gastroenterology 145, 1334–1346.e1–11. 10.1053/j.gastro.2013.08.017 23958540

[B23] Catalán M. A. Flores C. A. González-Begne M. Zhang Y. Sepúlveda F. V. Melvin J. E. (2012). Severe defects in absorptive ion transport in distal colons of mice that lack ClC-2 channels. Gastroenterology 142, 346–354. 10.1053/j.gastro.2011.10.037 22079595 PMC3267842

[B24] Chey W. D. Camilleri M. Chang L. Rikner L. Graffner H. (2011). A randomized placebo-controlled phase IIb trial of a3309, a bile acid transporter inhibitor, for chronic idiopathic constipation. Am. J. Gastroenterol. 106, 1803–1812. 10.1038/ajg.2011.162 21606974 PMC3188811

[B25] Chey W. D. Webster L. Sostek M. Lappalainen J. Barker P. N. Tack J. (2014). Naloxegol for opioid-induced constipation in patients with noncancer pain. N. Engl. J. Med. 370, 2387–2396. 10.1056/NEJMoa1310246 24896818

[B26] Chu H. Zhong L. Li H. Zhang X. Zhang J. Hou X. (2014). Epidemiology characteristics of constipation for general population, pediatric population, and elderly population in china. Gastroenterol. Res. Pract. 2014:532734. 10.1155/2014/532734 25386187 PMC4216714

[B27] Cil O. Phuan P. W. Lee S. Tan J. Haggie P. M. Levin M. H.. (2016). CFTR activator increases intestinal fluid secretion and normalizes stool output in a mouse model of constipation. Cell Mol. Gastroenterol. Hepatol. 2, 317–327. 10.1016/j.jcmgh.2015.12.010 27127798 PMC4844355

[B28] Cil O. Phuan P. W. Son J. H. Zhu J. S. Ku C. K. Tabib N. A.. (2017). Phenylquinoxalinone CFTR activator as potential prosecretory therapy for constipation. Transl. Res. 182, 14–26.e4. 10.1016/j.trsl.2016.10.003 27815136 PMC5453637

[B29] Coates M. D. Mahoney C. R. Linden D. R. Sampson J. E. Chen J. Blaszyk H.. (2004). Molecular defects in mucosal serotonin content and decreased serotonin reuptake transporter in ulcerative colitis and irritable bowel syndrome. Gastroenterology 126, 1657–1664. 10.1053/j.gastro.2004.03.013 15188158

[B30] Corsetti M. Tack J. (2015a). Naloxegol: the first orally administered, peripherally acting, mu opioid receptor antagonist, approved for the treatment of opioid-induced constipation. Drugs Today (Barc) 51, 479–489. 10.1358/dot.2015.51.8.2364896 26380386

[B31] Corsetti M. Tack J. (2015b). Naloxegol, a new drug for the treatment of opioid-induced constipation. Expert. Opin. Pharmacother. 16, 399–406. 10.1517/14656566.2015.991306 25496063

[B32] Costedio M. M. Coates M. D. Brooks E. M. Glass L. M. Ganguly E. K. Blaszyk H. (2010). Mucosal serotonin signaling is altered in chronic constipation but not in opiate-induced constipation. Am. J. Gastroenterol. 105, 1173–1180. 10.1038/ajg.2009.683 20010921 PMC2872481

[B33] Costilla V. C. Foxx-Orenstein A. E. (2014). Constipation: understanding mechanisms and management. Clin. Geriatr. Med. 30, 107–115. 10.1016/j.cger.2013.10.001 24267606

[B34] Cryer B. Katz S. Vallejo R. Popescu A. Ueno R. (2014). A randomized study of lubiprostone for opioid-induced constipation in patients with chronic noncancer pain. Pain Med. 15, 1825–1834. 10.1111/pme.12437 24716835 PMC4282321

[B35] Cuppoletti J. Malinowska D. H. Tewari K. P. Li Q. J. Sherry A. M. Patchen M. L.. (2004). SPI-0211 activates T84 cell chloride transport and recombinant human ClC-2 chloride currents. Am. J. Physiol. Cell Physiol. 287, C1173–1183. 10.1152/ajpcell.00528.2003 15213059

[B36] El-Salhy M. (2015). Recent developments in the pathophysiology of irritable bowel syndrome. World J. Gastroenterol. 21, 7621–7636. 10.3748/wjg.v21.i25.7621 26167065 PMC4491952

[B37] Eutamene H. Bradesi S. Larauche M. Theodorou V. Beaufrand C. Ohning G.. (2010). Guanylate cyclase C-mediated antinociceptive effects of linaclotide in rodent models of visceral pain. Neurogastroenterol. Motil. 22, 312–e84. 10.1111/j.1365-2982.2009.01385.x 19706070

[B38] Fei G. Raehal K. Liu S. Qu M. H. Sun X. Wang G. D.. (2010). Lubiprostone reverses the inhibitory action of morphine on intestinal secretion in guinea pig and mouse. J. Pharmacol. Exp. Ther. 334, 333–340. 10.1124/jpet.110.166116 20406855 PMC2912047

[B39] Fock K. M. Wagner A. (2007). Safety, tolerability and satisfaction with tegaserod therapy in Asia-Pacific patients with irritable bowel syndrome with constipation. J. Gastroenterol. Hepatol. 22, 1190–1198. 10.1111/j.1440-1746.2007.04955.x 17524039

[B40] Gershon M. D. Tack J. (2007). The serotonin signaling system: from basic understanding to drug development for functional GI disorders. Gastroenterology 132, 397–414. 10.1053/j.gastro.2006.11.002 17241888

[B41] Goldberg M. Li Y. P. Johanson J. F. Mangel A. W. Kitt M. Beattie D. T.. (2010). Clinical trial: the efficacy and tolerability of velusetrag, a selective 5-HT4 agonist with high intrinsic activity, in chronic idiopathic constipation—a 4-week, randomized, double-blind, placebo-controlled, dose-response study. Aliment. Pharmacol. Ther. 32, 1102–1112. 10.1111/j.1365-2036.2010.04456.x 21039672

[B42] Guay D. R. (2009). Methylnaltrexone methobromide: the first peripherally active, centrally inactive opioid receptor-antagonist. Consult. Pharm. 24, 210–226. 10.4140/TCP.n.2009.210 19555136

[B43] Günther T. Dasgupta P. Mann A. Miess E. Kliewer A. Fritzwanker S.. (2017). Targeting multiple opioid receptors - improved analgesics with reduced side effects? Br. J. Pharmacol. [Epub ahead of print]. 10.1111/bph.13809 28378462 PMC6016677

[B44] He C. L. Soffer E. E. Ferris C. D. Walsh R. M. Szurszewski J. H. Farrugia G. (2001). Loss of interstitial cells of cajal and inhibitory innervation in insulin-dependent diabetes. Gastroenterology, 121, 427–434. 10.1053/gast.2001.26264 11487552

[B45] Hoekman D. R. Benninga M. A. (2013). Functional constipation in childhood: current pharmacotherapy and future perspectives. Expert. Opin. Pharmacother. 14, 41–51. 10.1517/14656566.2013.752816 23216375

[B46] Hwang S. J. Blair P. J. Britton F. C. O'Driscoll K. E. Hennig G. Bayguinov Y. R.. (2009). Expression of anoctamin 1/TMEM16A by interstitial cells of Cajal is fundamental for slow wave activity in gastrointestinal muscles. J. Physiol. 587, 4887–4904. 10.1113/jphysiol.2009.176198 19687122 PMC2770154

[B47] Ironwood Pharmaceuticals Inc (2011a). Treatments for Gastrointestinal Disorders. WO103311 (A3). Cambridge: Ironwood Pharmaceuticals Inc.

[B48] Ironwood Pharmaceuticals Inc (2011b). Treatment of Chronic Constipation. WO056850 (A3). Cambridge: Ironwood Pharmaceuticals Inc.

[B49] Ironwood Pharmaceuticals Inc (2013). Treatments for Gastrointestinal Disorders. WO025969 (A1). Cambridge: Ironwood Pharmaceuticals Inc.

[B50] Ironwood Pharmaceuticals Inc (2015). Low-dose Stable Formulations of Linaclotide. WO089335 (A1). Cambridge: Ironwood Pharmaceuticals Inc.

[B51] Jakab R. L. Collaco A. M. Ameen N. A. (2012). Lubiprostone targets prostanoid signaling and promotes ion transporter trafficking, mucus exocytosis, and contractility. Dig. Dis. Sci. 57, 2826–2845. 10.1007/s10620-012-2352-8 22923315 PMC3482986

[B52] Jamal M. M. Adams A. B. Jansen J. P. Webster L. R. (2015). A randomized, placebo-controlled trial of lubiprostone for opioid-induced constipation in chronic noncancer pain. Am. J. Gastroenterol. 110, 725–732. 10.1038/ajg.2015.106 25916220 PMC4424379

[B53] Jiang C. Xu Q. Wen X. Sun H. (2015). Current developments in pharmacological therapeutics for chronic constipation. Acta. Pharm. Sin. B. 5, 300–309. 10.1016/j.apsb.2015.05.006 26579459 PMC4629408

[B54] Jiao H. Y. Kim D. H. Ki J. S. Ryu K. H. Choi S. Jun J. Y. (2014). Effects of lubiprostone on pacemaker activity of interstitial cells of cajal from the mouse colon. Korean J. Physiol. Pharmacol. 18, 341–346. 10.4196/kjpp.2014.18.4.341 25177167 PMC4146637

[B55] Johanson J. F. Drossman D. A. Panas R. Wahle A. Ueno R. (2008a). Clinical trial: phase 2 study of lubiprostone for irritable bowel syndrome with constipation. Aliment. Pharmacol. Ther. 27, 685–696. 10.1111/j.1365-2036.2008.03629.x 18248656

[B56] Johanson J. F. Morton D. Geenen J. Ueno R. (2008b). Multicenter, 4-week, double-blind, randomized, placebo-controlled trial of lubiprostone, a locally-acting type-2 chloride channel activator, in patients with chronic constipation. Am. J. Gastroenterol. 103, 170–177. 10.1111/j.1572-0241.2007.01524.x 17916109

[B57] Johnston J. M. Kurtz C. B. Macdougall J. E. Lavins B. J. Currie M. G. Fitch D. A.. (2010). Linaclotide improves abdominal pain and bowel habits in a phase IIb study of patients with irritable bowel syndrome with constipation. Gastroenterology 139, 1877–1886.e2. 10.1053/j.gastro.2010.08.041 20801122

[B58] Klein S. Seidler B. Kettenberger A. Sibaev A. Rohn M. Feil R.. (2013). Interstitial cells of Cajal integrate excitatory and inhibitory neurotransmission with intestinal slow-wave activity. Nat. Commun. 4:1630. 10.1038/ncomms2626 23535651

[B59] Knowles C. H. Farrugia G. (2011). Gastrointestinal neuromuscular pathology in chronic constipation. Best Pract. Res. Clin. Gastroenterol. 25, 43–57. 10.1016/j.bpg.2010.12.001 21382578 PMC4175481

[B60] Ko E. A. Jin B. J. Namkung W. Ma T. Thiagarajah J. R. Verkman A. S. (2014). Chloride channel inhibition by a red wine extract and a synthetic small molecule prevents rotaviral secretory diarrhoea in neonatal mice. Gut 63, 1120–1129. 10.1136/gutjnl-2013-305663 24052273 PMC4048772

[B61] Koch A. Voderholzer W. A. Klauser A. G. Müller-Lissner S. (1997). Symptoms in chronic constipation. Dis. Colon Rectum. 40, 902–906. 9269805 10.1007/BF02051196

[B62] Lacy B. E. Schey R. Shiff S. J. Lavins B. J. Fox S. M. Jia X. D.. (2015). Linaclotide in chronic idiopathic constipation patients with moderate to severe abdominal bloating: a randomized, controlled trial. PLoS ONE 10:e0134349. 10.1371/journal.pone.0134349 26222318 PMC4519259

[B63] Lembo A. J. Schneier H. A. Shiff S. J. Kurtz C. B. MacDougall J. E. Jia X. D.. (2011). Two randomized trials of linaclotide for chronic constipation. N. Engl. J. Med. 365, 527–536. 10.1056/NEJMoa1010863 21830967

[B64] Leonard J. Baker D. E. (2015). Naloxegol: treatment for opioid-induced constipation in chronic non-cancer pain. Ann. Pharmacother. 49, 360–365. 10.1177/1060028014560191 25471070

[B65] Leyva-Vega M. Gerfen J. Thiel B. D. Jurkiewicz D. Rand E. B. Pawlowska J.. (2010). Genomic alterations in biliary atresia suggest region of potential disease susceptibility in 2q37.3. Am. J. Med. Genet A 152A, 886–895. 10.1002/ajmg.a.33332 20358598 PMC2914625

[B66] Li F. Fu T. Tong W. D. Liu B. H. Li C. X. Gao Y.. (2016). Lubiprostone is effective in the treatment of chronic idiopathic constipation and irritable bowel syndrome: a systematic review and meta-analysis of randomized controlled trials. Mayo Clin. Proc. 91, 456–468. 10.1016/j.mayocp.2016.01.015 27046523

[B67] Liem O. Harman J. Benninga M. Kelleher K. Mousa H. Di Lorenzo C. (2009). Health utilization and cost impact of childhood constipation in the United States. J. Pediatr. 154, 258–262. 10.1016/j.jpeds.2008.07.060 18822430

[B68] Lonza A. G. (2014). Method for Preparation of Linaclotide. WO188011 (A3). Visp: Lonza A.G.

[B69] Manini M. L. Camilleri M. Goldberg M. Sweetser S. McKinzie S. Burton D.. (2010). Effects of Velusetrag (TD-5108) on gastrointestinal transit and bowel function in health and pharmacokinetics in health and constipation. Neurogastroenterol. Motil. 22, 42–9, e7–8. 10.1111/j.1365-2982.2009.01378.x 19691492 PMC2905526

[B70] McWilliams V. Whiteside G. McKeage K. (2010). Linaclotide: first global approval. Drugs 72, 2167–2175. 10.2165/11470590-000000000-00000 23083112

[B71] Mehta N. O'Connell K. Giambrone G. P. Baqai A. Diwan S. (2016). Efficacy of methylnaltrexone for the treatment of opiod-induced constipation: a meta-analysis and systematic review. Postgrad. Med. 128, 282–289. 10.1080/00325481.2016.1149017 26839023

[B72] Menees S. Saad R. Chey W. D. (2012). Agents that act luminally to treat diarrhoea and constipation. Nat. Rev. Gastroenterol. Hepatol. 9, 661–674. 10.1038/nrgastro.2012.162 22945441

[B73] Mertz H. Naliboff B. Mayer E. (1999). Physiology of refractory chronic constipation. Am. J. Gastroenterol. 94, 609–615. 10.1111/j.1572-0241.1999.922_a.x 10086639

[B74] Mugie S. M. Di Lorenzo C. Benninga M. A. (2011). Constipation in childhood. Nat. Rev. Gastroenterol. Hepatol. 8, 502–511. 10.1038/nrgastro.2011.130 21808283

[B75] Namkung W. Yao Z. Finkbeiner W. E. Verkman A. S. (2011). Small-molecule activators of TMEM16A, a calcium-activated chloride channel, stimulate epithelial chloride secretion and intestinal contraction. FASEB J. 25, 4048–4062. 10.1096/fj.11-191627 21836025 PMC3205834

[B76] Nelson A. D. Camilleri M. (2016). Opioid-induced constipation: advances and clinical guidance. Ther. Adv. Chronic. Dis. 7, 121–134. 10.1177/2040622315627801 26977281 PMC4772344

[B77] Norimatsu Y. Moran A. R. MacDonald K. D. (2012). Lubiprostone activates CFTR, but not ClC-2, via the prostaglandin receptor (EP(4)). Biochem. Biophys. Res. Commun. 426, 374–379. 10.1016/j.bbrc.2012.08.097 22960173 PMC3489164

[B78] Nyam D. C. Pemberton J. H. Ilstrup D. M. Rath D. M. (1997). Long-term results of surgery for chronic constipation. Dis. Colon. Rectum. 40, 273–279. 9118740 10.1007/BF02050415

[B79] Purdue Pharma Lp (2012). Buprenorphine Analogs. WO038813(A1). Stamford: Purdue Pharma Lp.

[B80] Purdue Pharma Lp (2013a). Benzomorphan Compounds as Opioid Receptors Modulators. WO167963 (A1). Stamford: Purdue Pharma Lp.

[B81] Purdue Pharma Lp (2013b). Quaternized Buprenorphine Analogs. WO084060 (A1). Stamford: Purdue Pharma Lp.

[B82] Purdue Pharma Lp (2014a). Lockman Jeffrey. Benzomorphan Analogs and the Use Thereof. WO072809 (A3). Stamford: Purdue Pharma Lp.

[B83] Purdue Pharma Lp (2014b). Buprenorphine Analogs. WO140784(A3). Stamford: Purdue Pharma Lp.

[B84] Purdue Pharma Lp (2014c). Buprenorphine Analogs. WO118618(A1). Stamford: Purdue Pharma Lp.

[B85] Purdue Pharma Lp (2015). Benzomorphan Analogs and Use Thereof. WO171553. Stamford: Purdue Pharma Lp.

[B86] Qilu Pharmaceutical Co Ltd (2015a). Novel Crystal Form of Lubiprostone and Preparation Method of Crystal Form. CN104710398 (A). Jinan: Qilu Pharmaceutical Co Ltd.

[B87] Qilu Pharmaceutical Co Ltd (2015b). Method for Preparing Lubiprostone Compound. CN104557845 (A). Jinan: Qilu Pharmaceutical Co Ltd.

[B88] Quigley E. M. Tack J. Chey W. D. Rao S. S. Fortea J. Falques M.. (2013). Randomised clinical trials: linaclotide phase 3 studies in IBS-C - a prespecified further analysis based on European Medicines Agency-specified endpoints. Aliment. Pharmacol. Ther. 37, 49–61. 10.1111/apt.12123 23116208

[B89] Quigley E. M. Wald A. Fidelholtz J. Boivin M. Pecher E. Earnest D. (2006). Safety and tolerability of tegaserod in patients with chronic constipation: pooled data from two phase III studies. Clin. Gastroenterol. Hepatol. 4, 605–613. 10.1016/j.cgh.2006.02.017 16678076

[B90] Rao S. S. (2003). Constipation: evaluation and treatment. Gastroenterol. Clin. North Am. 32, 659–683. 10.1016/S0889-8553(03)00026-8 12858610

[B91] Rao S. S. Quigley E. M. Shiff S. J. Lavins B. J. Kurtz C. B. MacDougall J. E.. (2014). Effect of linaclotide on severe abdominal symptoms in patients with irritable bowel syndrome with constipation. Clin. Gastroenterol. Hepatol. 12, 616–623. 10.1016/j.cgh.2013.09.022 24075889

[B92] Rao S. S. Welcher K. D. Leistikow J. S. (1998). Obstructive defecation: a failure of rectoanal coordination. Am. J. Gastroenterol. 93, 1042–1050. 10.1111/j.1572-0241.1998.00326.x 9672327

[B93] Reddy's Lab Ltd (2016). Improved Process for Preparation of Amorphous Linaclotide. WO012938 (A3). Hyderabad: Reddy's Lab Ltd.

[B94] Rey E. Balboa A. Mearin F. (2014). Chronic constipation, irritable bowel syndrome with constipation and constipation with pain/discomfort: similarities and differences. Am. J. Gastroenterol. 109, 876–884. 10.1038/ajg.2014.18 24589666

[B95] Sajid M. S. Hebbar M. Baig M. K. Li A. Philipose Z. (2016). Use of prucalopride for chronic constipation: a systematic review and meta-analysis of published randomized, controlled trials. J. Neurogastroenterol. Motil. 22, 412–422. 10.5056/jnm16004 27127190 PMC4930296

[B96] Sanchez M. I. Bercik P. (2011). Epidemiology and burden of chronic constipation. Can. J. Gastroenterol. 25 (Suppl. B), 11B–15B. 10.1155/2011/974573 22114752 PMC3206560

[B97] Sanders K. M. Ward S. M. (2006). Interstitial cells of Cajal: a new perspective on smooth muscle function. J. Physiol. 576, 721–726. 10.1113/jphysiol.2006.115279 16873406 PMC1890422

[B98] Sarosiek I. Bashashati M. Alvarez A. Hall M. Shankar N. Gomez Y.. (2016). Lubiprostone accelerates intestinal transit and alleviates small intestinal bacterial overgrowth in patients with chronic constipation. Am. J. Med. Sci. 352, 231–238. 10.1016/j.amjms.2016.05.012 27650225

[B99] Scinopharm Kunshan Biochemical Technology Co Ltd (2015). Processes for Preparation of Lubiprostone. JP120693 (A). Kunshan: Scinopharm Kunshan Biochemical Technology Co Ltd.

[B100] Shailubhai K. Comiskey S. Foss J. A. Feng R. Barrow L. Comer G. M.. (2013). Plecanatide, an oral guanylate cyclase C agonist acting locally in the gastrointestinal tract, is safe and well-tolerated in single doses. Dig. Dis. Sci. 58, 2580–2586. 10.1007/s10620-013-2684-z 23625291

[B101] Shenzhen Hybio Pharmaceutical (2013a). Preparation Method of Plecanatide. CN103694320 (B). Shenzhen: Shenzhen Hybio Pharmaceutical.

[B102] Shenzhen Hybio Pharmaceutical (2013b). Preparation Method of Plecanatide. CN104211777 (A). Shenzhen: Shenzhen Hybio Pharmaceutical.

[B103] Sherid M. Sifuentes H. Samo S. Deepak P. Sridhar S. (2013). Lubiprostone induced ischemic colitis. World J. Gastroenterol. 19, 299–303. 10.3748/wjg.v19.i2.299 23345954 PMC3547564

[B104] Siemens W. Becker G. (2016). Methylnaltrexone for opioid-induced constipation: review and meta-analyses for objective plus subjective efficacy and safety outcomes. Ther. Clin. Risk Manag. 12, 401–412. 10.2147/TCRM.S80749 27042082 PMC4795449

[B105] Singh R. D. Gibbons S. J. Saravanaperumal S. A. Du P. Hennig G. W. Eisenman S. T.. (2014). Ano1, a Ca^2+^-activated Cl- channel, coordinates contractility in mouse intestine by Ca^2+^ transient coordination between interstitial cells of Cajal. J. Physiol. 592, 4051–4068. 10.1113/jphysiol.2014.277152 25063822 PMC4198014

[B106] Soubra M. Schey R. (2012). Lubiprostone for the treatment of adult women with irritable bowel syndrome with constipation. Clin. Med. Insights Gastroenterol. 5, 23–30. 10.4137/CGast.S7625 24833931 PMC3987758

[B107] Spierings E. L. Brewer R. P. Rauck R. L. Losch-Beridon T. Mareya S. M. (2017). Lubiprostone for opioid-induced constipation does not interfere with opioid analgesia in patients with chronic noncancer pain. Pain Pract. 17, 312–319. 10.1111/papr.12444 26990171

[B108] Spierings E. L. Rauck R. Brewer R. Marcuard S. Vallejo R. (2015). Long-term safety and efficacy of lubiprostone in opioid-induced constipation in patients with chronic noncancer pain. Pain Pract. 16, 985–993. 10.1111/papr.12347 26328775

[B109] Sun Pharmaceutical Ind Ltd (2016). Linaclotide Stable Composition. WO024291(A1). Maharashtra: Sun Pharmaceutical Ind Ltd.

[B110] Tack J. Camilleri M. Dubois D. Vandeplassche L. Joseph A. Kerstens R. (2015). Association between health-related quality of life and symptoms in patients with chronic constipation: an integrated analysis of three phase 3 trials of prucalopride. Neurogastroenterol. Motil. 27, 397–405. 10.1111/nmo.12505 25581251

[B111] Tack J. Corsetti M. (2014a). Naloxegol for the treatment of opioid-induced constipation. Expert. Rev. Gastroenterol. Hepatol. 8, 855–861. 10.1586/17474124.2014.939629 25220391

[B112] Tack J. Stanghellini V. Dubois D. Joseph A. Vandeplassche L. Kerstens R. (2014b). Effect of prucalopride on symptoms of chronic constipation. Neurogastroenterol. Motil. 26, 21–27. 10.1111/nmo.12217 24106924 PMC4282451

[B113] Tack J. van Outryve M. Beyens G. Kerstens R. Vandeplassche L. (2009). Prucalopride (Resolor) in the treatment of severe chronic constipation in patients dissatisfied with laxatives. Gut 58, 357–365. 10.1136/gut.2008.162404 18987031

[B114] Thomas R. H. Allmond K. (2013). Linaclotide (Linzess) for irritable bowel syndrome with constipation and for chronic idiopathic constipation. P. T. 38, 154–160. 23641133 PMC3638410

[B115] Thomas R. H. Luthin D. R. (2015). Current and emerging treatments for irritable bowel syndrome with constipation and chronic idiopathic constipation: focus on prosecretory agents. Pharmacotherapy 35, 613–630. 10.1002/phar.1594 26016701

[B116] Tien X. Y. Brasitus T. A. Kaetzel M. A. Dedman J. R. Nelson D. J. (1994). Activation of the cystic fibrosis transmembrane conductance regulator by cGMP in the human colonic cancer cell line, Caco-2. J. Biol. Chem. 269, 51–54. 7506258

[B117] Tougas G. Snape W. J. Jr. Otten M. H. Earnest D. L. Langaker K. E. Pruitt R. E.. (2002). Long-term safety of tegaserod in patients with constipation-predominant irritable bowel syndrome. Aliment. Pharmacol. Ther. 16, 1701–1708. 10.1046/j.1365-2036.2002.01347.x 12269961

[B118] Univ Nanjing Tech (2015). Preparation Method of Plecanatide. CN104628827 (A). Nanjing: Univ Nanjing Tech.

[B119] Vazquez Roque M. Bouras E. P. (2015). Epidemiology and management of chronic constipation in elderly patients. Clin. Interv. Aging 10, 919–930. 10.2147/CIA.S54304 26082622 PMC4459612

[B120] Wang H. L. (2015). Understanding the pathogenesis of slow-transit constipation: one step forward. Dig. Dis. Sci. 60, 2216–2218. 10.1007/s10620-015-3754-1 26088370

[B121] Wilson N. Schey R. (2015). Lubiprostone in constipation: clinical evidence and place in therapy. Ther. Adv. Chronic. Dis. 6, 40–50. 10.1177/2040622314567678 25729555 PMC4331234

[B122] Wong B. S. Camilleri M. McKinzie S. Burton D. Graffner H. Zinsmeister A. R. (2010). Effects of A3309, an ileal bile acid transporter inhibitor, on colonic transit and symptoms in females with functional constipation. Am. J. Gastroenterol. 106, 2154–2164. 10.1038/ajg.2011.285 21876564

[B123] Yao Z. Namkung W. Ko E. A. Park J. Tradtrantip L. Verkman A. S. (2012). Fractionation of a herbal antidiarrheal medicine reveals eugenol as an inhibitor of Ca^2+^-Activated Cl^−^ channel TMEM16A. PLoS ONE 7:e38030. 10.1371/journal.pone.0038030 22666439 PMC3364195

